# Effects of parental acclimation and energy limitation in response to high CO_2_ exposure in Atlantic cod

**DOI:** 10.1038/s41598-018-26711-y

**Published:** 2018-05-29

**Authors:** M. H. Stiasny, F. H. Mittermayer, G. Göttler, C. R. Bridges, I.-B. Falk-Petersen, V. Puvanendran, A. Mortensen, T. B. H. Reusch, C. Clemmesen

**Affiliations:** 1 GEOMAR Helmholtz Centre for Ocean Research Kiel, Evolutionary Ecology of Marine Fishes, Düsternbrooker Weg 20, 24105 Kiel, Germany; 2 University of Kiel, Department of Economics, Wilhelm-Seelig-Platz 1, 24118 Kiel, Germany; 3 Heinrich-Heine Universität Düsseldorf, Institute of Metabolic Physiology, 40225 Düsseldorf, Germany; 4 University of Tromsø, Faculty of Biosciences, Fisheries and Economics, Tromsø, Norway; 5 Nofima AS, Postboks 6122, NO-9291 Tromsø, Norway

## Abstract

Ocean acidification (OA), the dissolution of excess anthropogenic carbon dioxide in ocean waters, is a potential stressor to many marine fish species. Whether species have the potential to acclimate and adapt to changes in the seawater carbonate chemistry is still largely unanswered. Simulation experiments across several generations are challenging for large commercially exploited species because of their long generation times. For Atlantic cod (*Gadus morhua*), we present first data on the effects of parental acclimation to elevated aquatic CO_2_ on larval survival, a fundamental parameter determining population recruitment. The parental generation in this study was exposed to either ambient or elevated aquatic CO_2_ levels simulating end-of-century OA levels (~1100 µatm CO_2_) for six weeks prior to spawning. Upon fully reciprocal exposure of the F1 generation, we quantified larval survival, combined with two larval feeding regimes in order to investigate the potential effect of energy limitation. We found a significant reduction in larval survival at elevated CO_2_ that was partly compensated by parental acclimation to the same CO_2_ exposure. Such compensation was only observed in the treatment with high food availability. This complex 3-way interaction indicates that surplus metabolic resources need to be available to allow a transgenerational alleviation response to ocean acidification.

## Introduction

Atlantic cod (*Gadus morhua*) supports large, commercial fisheries in many areas of the Northern Atlantic. Different stocks are adapted to a wide range of temperatures and are predicted to react differently to changing temperatures, depending on where they already exist in relation to their thermal optimum^1^. The most Northern stocks are currently benefiting from ocean warming^2^ through range expansion and through direct positive effects of increasing temperatures on recruitment and growth^1^. Along with global warming, however, another inevitable and direct effect of CO_2_ emissions through human activity is ocean acidification, the dissolution of excess CO_2_ in ocean waters. The rate of ocean acidification is predicted to be highest in the Arctic ocean^3^, while the Arctic is also warming faster than the global average^4^, which puts Arctic species at a higher risk to be negatively affected by climate change than more southerly species. Given the mounting evidence for negative CO_2_ effects on fish early life stages^5–12^ any expectations that global warming might have positive effects on the fisheries in these areas^2^ is therefore premature. While some progress has been made in research on the potential for acclimation and adaptation in other taxa in response to acidification^13,14^, the role of transfer of information via transgenerational effects other than changing the heritable information of DNA sequence as a mechanism to accommodate climate change at the individual level (also called transgenerational plasticity or acclimation) is still under debate.

Only a handful of studies have recently addressed whether and how much transgenerational acclimation might affect fitness-relevant traits or population limiting rates of offspring, such as survival or growth, in response to ocean acidification. Most evidence of positive transgenerational effects comes from tropical reef fish, namely the Spiny Chromis (*Acanthochromis polyacanthus*)^15,16^ and the Fire Clownfish (*Amphiprion melanopus*)^17^, although Welch *et al*.^16^ showed no capacity for transgenerational capacity for olfactory responses to ocean acidification in spiny damselfish. However, in summary Rummer and Munday (2017)^18^ conclude that there is evidence of transgenerational acclimation and adaptation in response to ocean acidification and temperature. For temperate fish species, physiological responses to parental acclimation in stickleback exist^19–21^. Schade *et al*.^19^ demonstrated reduced survival and body size at 30dph and enlarged otoliths when fathers or both parents were acclimated to the high CO_2_ level. Shama & Wegner (2014)^20^ demonstrated that the reproductive output was mainly determined by the temperature the mother experienced with a carry-over effect from the grandmother. In older stages, maternal and maternal grandmother environments influenced stickleback’s body size, but in opposing directions, indicating that the mechanisms in the transfer of environmental information differed between the generations^20^. Acclimation of mothers to higher temperatures led to a more efficient offspring mitochondrial respiratory capacity, also reflected in the expression of the relevant genes (Shama *et al*.)^21^. Exploring the potential of fish populations to adapt to ocean acidification through multi-generational experiments similar to work in coral reef fishes^22^ and in stickleback, is unfeasible for most commercial species because of their long generation times (e.g. Atlantic cod 3–5 years) and larger body size. Assessing the effect of parental acclimation is therefore the closest current research can come to assess transgenerational effects. This research impasse is all the more troubling, because temperate to boreal species with commercial importance can be remarkably sensitive to high CO_2_^5,6^. For example, our group has previously shown in separate experiments that Atlantic cod stocks from the Western Baltic and the Barents Sea consistently suffered from higher mortality rates under realistic, end-of-century levels of ocean acidification, with the salient finding that daily mortality rates doubled in both stocks^8^.

Here, we exposed adult cod to either ambient seawater or seawater with increased aquatic CO_2_ concentrations, and therefore pH changes, for six weeks prior to spawning. We used ~1100 µatm as the high CO_2_ treatment, which is expected globally around the year 2100 following the scenario IPCC RCP 8.5^4^. However, local predictions show that this level of acidification will likely be reached in the Arctic under the RCP 4.5 or at even lower emissions^23^. The exposure of the adult generation to CO_2_ coincides with the last stages of gonadal development and egg maturation^24^. Resulting eggs and larvae were reared either in the parental CO_2_ concentrations or the opposite treatment (i.e. high CO_2_ treatment in low CO_2_ and vice versa). Larval survival and growth were measured and histological samples of certain organs, including the eyes and the liver, were processed and analyzed. The tested hypothesis was that larvae, which came from parents, who already experienced exposure to high CO_2_ during gonadal development, might cope better with these conditions due to possible acclimation of the parents or because developing eggs in the mother experienced the high CO_2_ conditions.

Additionally, we tested the effect of energy limitation by including two different feeding regimes as additional treatments, fully crossed with the CO_2_ treatments. Acid-Base balance is a costly process in marine fishes. Hydrogen ions are expelled at the gills by an H^+^/Na^+^ exchanger. While this is a passive process, it is fuelled by the concentration gradient of sodium ions between the gill cell and the seawater, which is maintained by the K^+^/Na^+^ ATPase^25,26^. During continuous exposure to a high CO_2_ aquatic environment, this process needs to be constantly upregulated, resulting in an additional energetic cost to the organism^27^. This aspect of ocean acidification and its effect on acid-base balance has so far been largely ignored when studying fishes, though it is reasonable to assume that organisms, which are energy limited already, may respond differently to CO_2_ stress than those, which are not limited. We therefore hypothesized that larvae, which are fed *ad libitum* may be more resilient to the exposure to high CO_2_ than those on a lower feeding regime and that this might interact with the effect of parental acclimation to high CO_2_.

## Results

**Figure 1:**
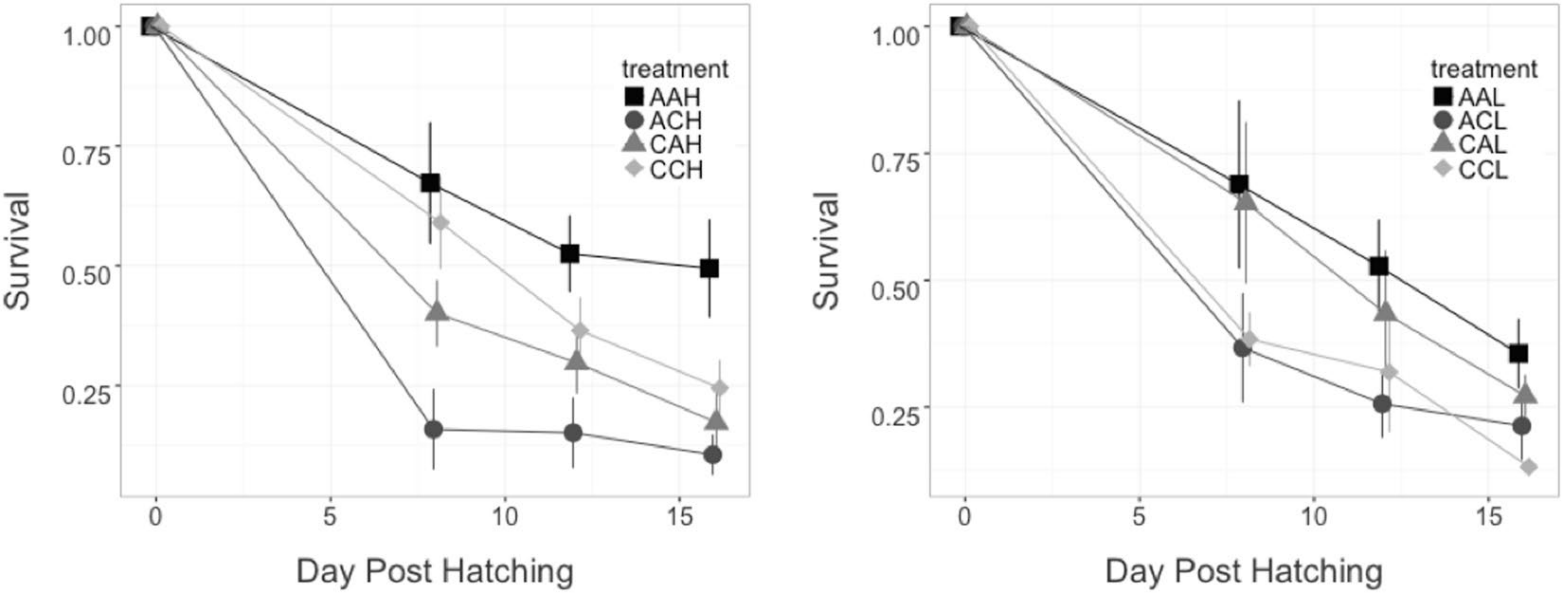
Survival of cod larvae from hatching to 16 days post-hatching in the high food treatment (left) and the low food treatment (right) depending on parental CO_2_, larval CO_2_ treatment and food. Shown are mean values and standard error across three replicates per treatment. The first letter of the legend refers to the parental CO_2_ treatment, the second to the larval CO_2_ treatment and the third to the food treatment. (A-Ambient, C-High CO_2_, H-High Food, L-Low Food).

In this study, the parental exposure to high CO_2_ modified the physiological reaction of larvae in the subsequent generation. This furthermore depended on the food availability, as shown by the significant three-way interaction of larval CO_2_, parental CO_2_ and food treatment (Table 1). Offspring in the high CO_2_ treatment of parents that were exposed to high CO_2_ survived better under high food availability (24.5% on day 16) compared to offspring from non-acclimated parents (10.5%), but worse under low food availability (on average 13.2% on day 16, compared to 21.3%) (Fig. 1). In the high food treatment, larvae of parents acclimated to high CO_2_ under high CO_2_ showed survival intermediate between the ambient CO_2_ treatment (49.4% on the final sampling day) and those without prior exposure in the parental generation to increased acidification (10.5%). This compensation was completely missing in the low food treatment. Here, larvae exposed to high CO_2_ coming from CO_2_ acclimated parents showed even lower survival (13.2%) than those from non-acclimated parents (21.3%). The survival of larvae in ambient CO_2_ conditions, but from parents acclimated to high CO_2_ (27.2%), was lower than that of larvae under ambient conditions from parents under ambient conditions (35.6%), and slightly higher than that of larvae, who were exposed to high CO_2_, but whose parents came from ambient CO_2_ conditions (21.2%).

**Table 1:** Degrees of freedom, F-values and p-values of the statistical analyses of standard length, dry weight and survival depending on the treatment.

Tested parameter	Factor	df	F value	p value
Standard Length	Parental CO_2_ treatment	18	0.475	0.4996
	Larval CO_2_ treatment	18	1.115	0.3049
	Food treatment	**18**	**40.940**	**<.0001**
Dry Weight (log transformed)	Parental CO_2_ treatment	18	0.145	0.708
	Larval CO_2_ treatment	18	0.424	0.523
	Food treatment	**18**	**48.784**	**<0.0001**
Survival (logit transformed)	Parental CO_2_ treatment	1	0.021	0.885
	**Larval CO**_**2**_ **treatment**	**1**	**10.831**	0.002**
	**Food treatment**	**1**	**4.491**	0.039*
	Day	1	1.976	0.166
	**Parental CO**_**2**_ *Larval CO_**2**_ **Treatment**	**1**	**15.102**	0.0003***
	Parental CO_2_ *Food Treatment	1	0.793	0.377
	Larval CO_2_ *Food Treatment	1	0.121	0.73
	Parental CO_2_ Treatment *Day	1	0.819	0.37
	Larval CO_2_ Treatment *Day	1	0.171	0.68
	Food CO_2_ Treatment *Day	1	1.042	0.312
	**Parental CO**_**2**_ *Larval CO_**2**_ Treatment *Food Treatment	**1**	**8.171**	0.006**
	Parental CO_2_ *Larval CO_2_ Treatment *Day	1	0.902	0.347
	Parental CO_2_ *Food Treatment *Day	1	0.546	0.463
	Larval CO_2_ *Food Treatment *Day	1	0.523	0.473
	Parental CO_2_ *Larval CO_2_ *Food Treatment *Day	1	0.002	0.964

**Figure 2:**
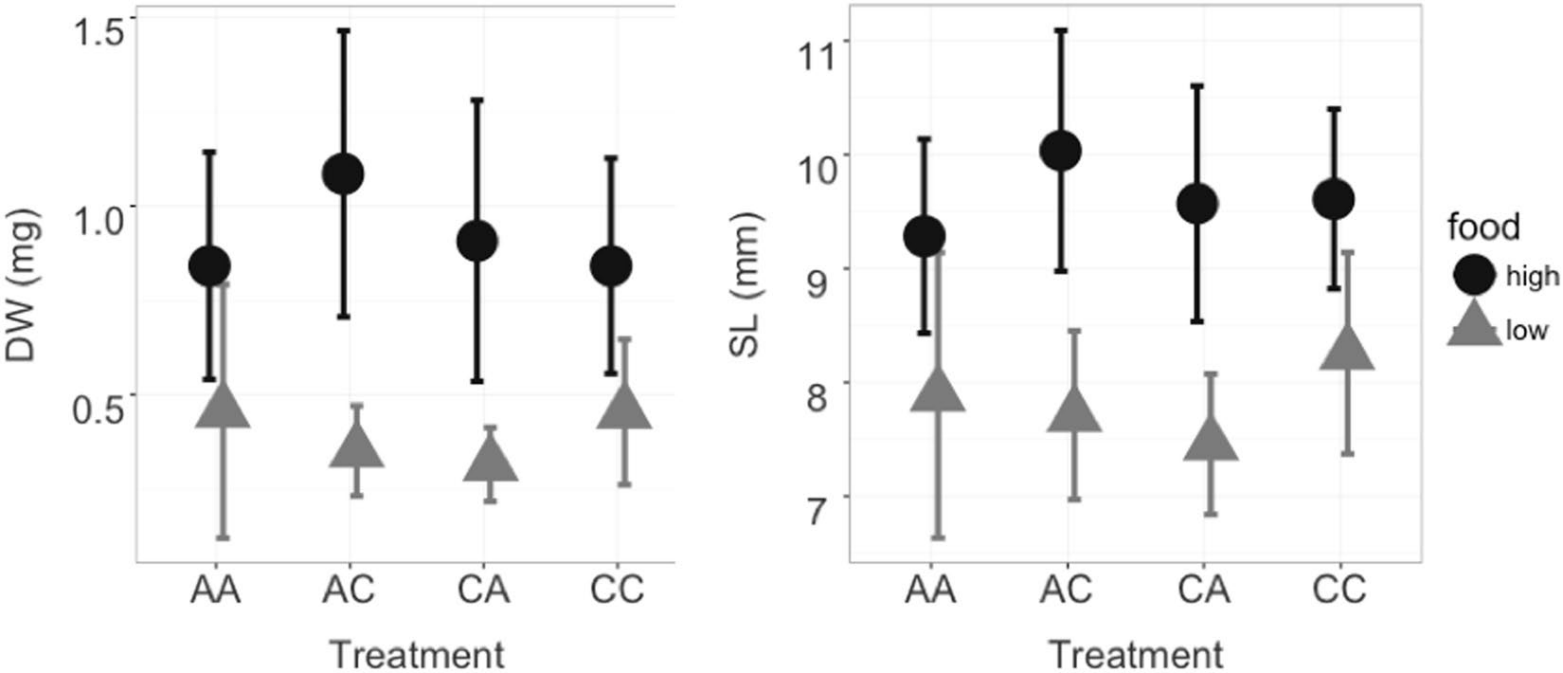
Dry weight (in mg, on the left) and Standard length (in mm, on the right) in 36 days post-hatching cod larvae depending on parental CO_2_ and larval CO_2_ treatment and food availability. Shown are mean values and standard deviation of ten larvae per three replicates. The first letter of the legend refers to the parental CO_2_ treatment and the second to the larval CO_2_ treatment. (A-Ambient, C-High CO_2_) High food is shown in dark circles and low food in lighter triangles.

Larval growth in terms of dry weight and standard length at day 36 post-hatching was not affected by the CO_2_ treatment of parents nor the offspring, but larvae in the low food treatment were smaller (Fig. 2), indicating that offspring growth was energy limited. Since the parental generation is a F3 aquaculture stock, bred for optimal growth, it is unlikely that this absence of an effect of exposure to high CO_2_ is directly transferable in wild populations.

**Figure 3:**
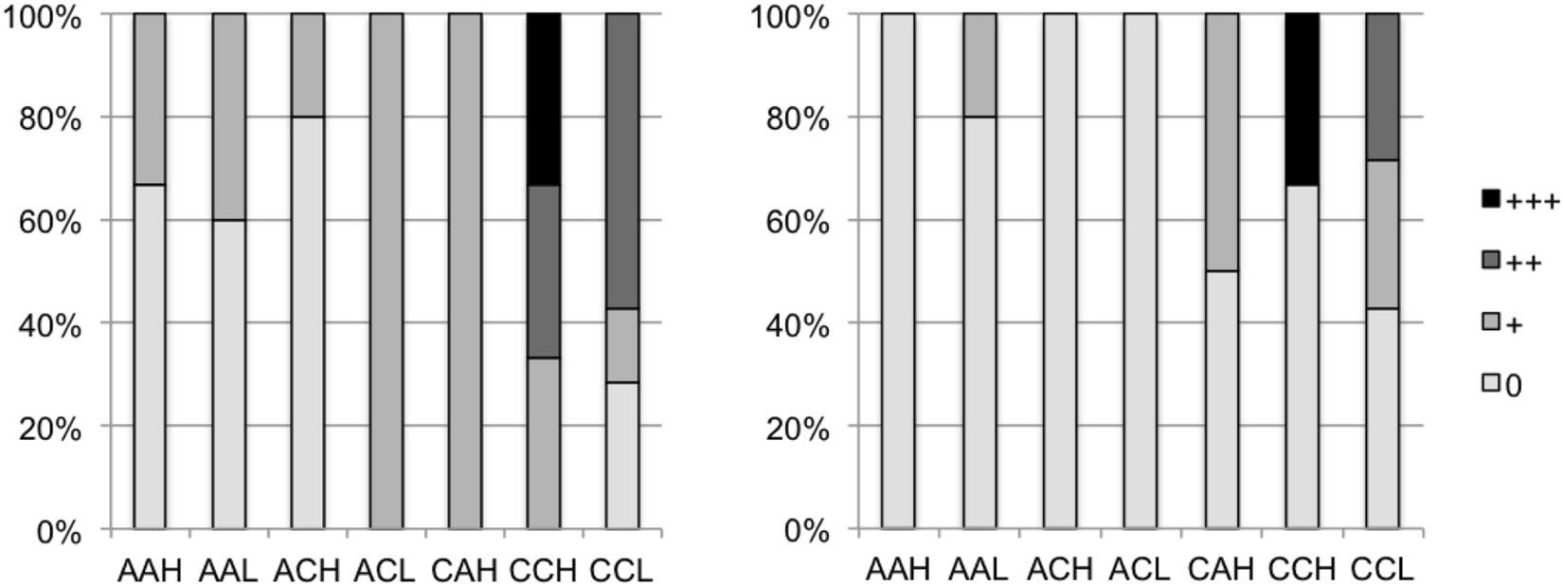
Frequency of liver (left) and eye (right) vacuolization in subjective scores from 0 to +++ depending on parental treatment (1^st^ letter (A-Ambient, C-high CO_2_)), larval CO_2_ treatment (2^nd^ letter (A-Ambient, C-high CO_2_)) and food treatment (3^rd^ letter, H – high food, L – low food). (N = 1–7)

**Figure 4:**
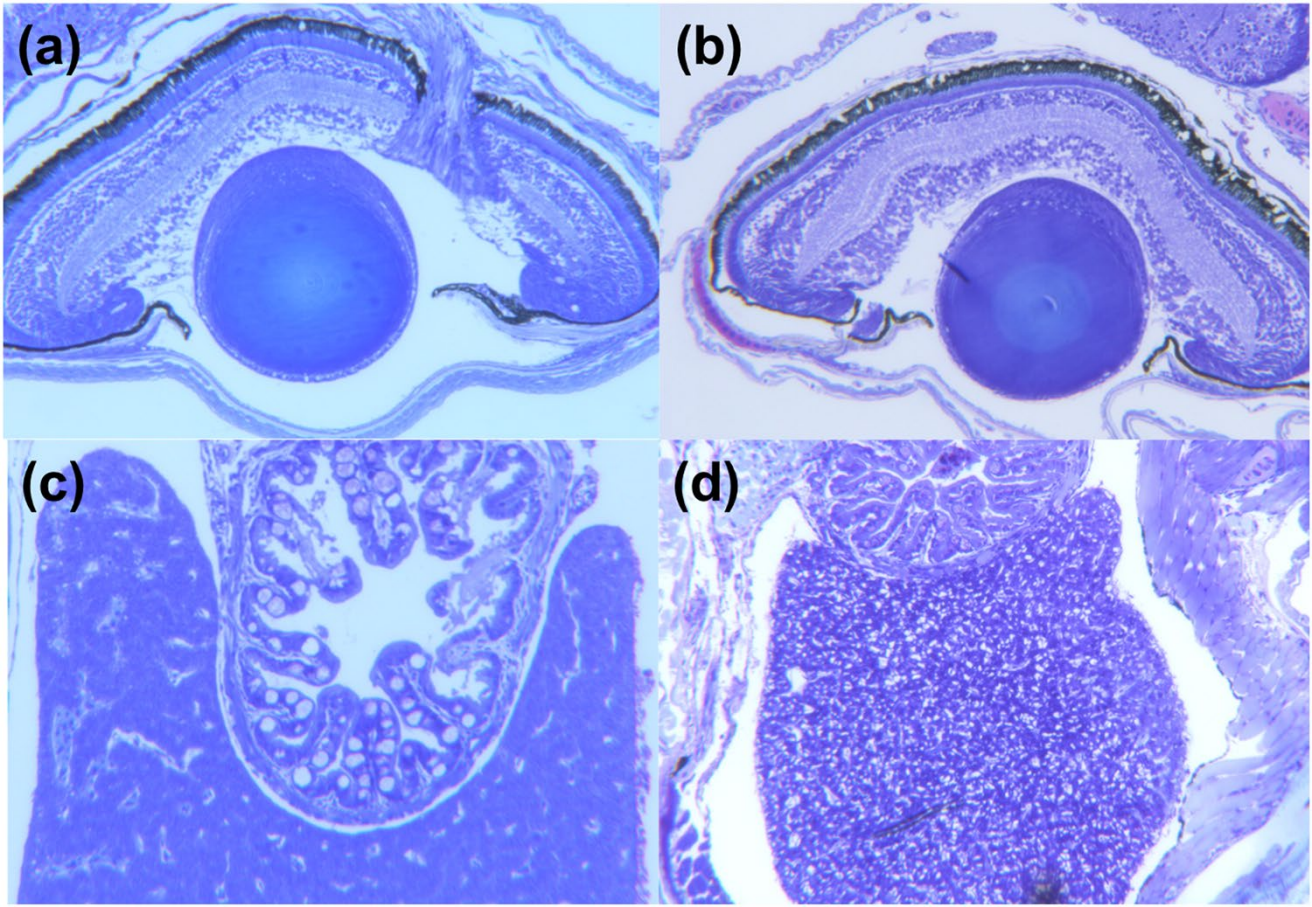
Examples of histological sections; (**a**) Transverse of eye with few vacuoles in the pigmented layer of the retina from AAL larva; (**b**) Transverse section of eye with many vacuoles in the pigmented layer of the retina from CCH larva; (**c**) Transverse section of liver and oesophagus of AAL larva. Note few vacuolizations in the liver; (**d**) Transverse section of liver and oesophagus of CCH larva. Note numerous vacuolizations in the liver.

Under CO_2_ concentrations corresponding to realistic end-of century ocean acidification levels, we found histological damage suggesting impairments of major organ functioning. Particularly the larvae in the high CO_2_ treatment, which came from acclimated parents, showed strong impairments more frequently independently of the food treatment (Figs 3 and 4). Vacuoles in the pigment layer of the retina of the 35 days old larvae were registered in all treatments but were more frequent in larvae from tanks with elevated CO_2_ concentrations (Fig. 3). Gill structure looked similar in all investigated larvae. Similar heart morphology was also noted in all larvae. The kidney tissue sections showed apparently normal tubuli and glomeruli in all groups. Liver morphology varied between individual samples and CO_2_ regimes (Fig. 4c,d). Glycogen granules were noted in all livers sectioned (Fig. 4c), while numerous empty vacuoles (representing lipid inclusions) of variable sizes were characteristic of some of the CO_2_ treated larvae (Fig. 4d). Such abnormal vacuolation will impair liver function^5,6^. In contrast, larvae from the ambient treatment had smaller and more regular vacuoles (Fig. 4c). Hepatocyte vacuolation did not occur more frequently in the larval group in the high food compared to the low food treatment.

## Discussion

In this study, we analyzed whether or not parental acclimation together with the corresponding exposure of gamete development in the parents to high aquatic CO_2_ conditions would result in improved performance of the next generation in a high CO_2_ environment. Our data were collected for a commercially exploited fish species, the Atlantic cod. In response to elevated CO_2_, in this independent new data set, cod larval mortality increased in accordance to observations previously described by Stiasny *et al*.^8^. Additionally, this study found that parental acclimation alleviated negative effects of high CO_2_ exposure on larval mortality to some degree, suggesting that such effects may change first order effects that only address physiological effects within the same generation^5–8^. However, this alleviation was only observed under high food availability, demonstrating the complexity of ocean acidification with other interacting factors, here food availability on fish physiology and survival. Therefore, knowledge of the food web interactions in response to ocean acidification has to be taken into consideration to predict how acclimation effects can potentially alleviate the so far observed direct negative effects of ocean acidification on larval fish survival and the long-term effects on stocks.

The potential energetic impact of ocean acidification on marine fish species has often been ignored in previous studies, except for a recent study by Bignami *et al*.^28^ showing a negative effect of elevated CO_2_ at complete feeding cessation on the starvation potential in larval cobia and a study of Hurst *et al*.^29^, which found no interaction between CO_2_ and nutritional stress in the northern rock sole^29^. Since it is well known that adult fish, juveniles and later larval stages are efficient at acid base regulation, they have often been assumed to be robust to ocean acidification, ignoring the fact that this comes at a high energetic cost and a changed acid base balance of the blood^25,27^. We were able to show for the first time that food limitation in fact has a large impact on the effect of high CO_2_ exposure and can significantly affect the fitness of cod larvae.

Considering that our simulated low food treatment is likely to be above common prey availabilities in the field, it is unlikely that fish larvae in the wild will have the necessary energy available to regulate efficiently throughout their development and, as also shown in this study, to benefit from parental acclimation to similar conditions as experienced by them. The study shows the importance of including energy availability in experimental studies of ocean acidification in the future.

Since the exposure time of the parental generation to high CO_2_ was only six weeks in duration, we are unable to distinguish between transgenerational effects in the strictest sense and an effect of exposure of the early zygotic development in the mother or father to CO_2_, a point well raised by Torda *et al*.^14^. However, our main aim was to explore whether the exposure of multiple life stages to increased CO_2_ environments would benefit the overall survival and therefore reproductive potential or not.

Our results show that parental exposure to high CO_2_ conditions results in reduced survival, when the larvae were raised under ambient CO_2_ concentrations, compared to those, where neither parents nor larvae experienced any level of acidification. Possibly, there is already a negative impact of high CO_2_ exposure on the eggs mediated via the mother during early zygotic development, which does result in lower fitness during later life stages.

The histological organ impairments found here are consistent with those found by Frommel *et al*.^5,6,11^. The degree of damage is lower, probably due to the more realistic, lower levels of carbon dioxide used in this experiment (1100 µatm in this study compared to 1800 and 4200 µatm by Frommel). The histological sections show that even in the high food treatment, where larval survival was partly compensated by the parental acclimation, larvae still suffered developmental impairments and organ damage in response to high CO_2_. This might suggest, that even though survival is slightly improved at this point by parental exposure to CO_2_, the long-term fitness of the larvae may very well suffer, since the impairments of the organs may result in later functional problems.

In conclusion, we found an effect of parental acclimation to CO_2_ exposure, however only under ideal conditions concerning prey availability, a situation unlikely to be expected in the wild. Our results highlight the importance of energy availability. This is adding to the uncertainty of effects of ocean acidification on marine fish, since acidification may well change ecosystems and the food web structures^30–32^, therefore altering prey availabilities.

## Methods

The experiments were performed at the Centre for Marine Aquaculture (*Senter for marin akvakultur*, formerly the Norwegian Cod Breeding Centre *Nasjonal avlsstasjon for torsk*) of Nofima outside of Tromsø, Norway during the spring of 2014. We used a full factorial experimental design combining high and low CO_2_ treatments to simulate ocean acidification and two different feeding treatments during the larval stage development.

### Water treatment

Deep-water from 40 to 60 m depths was pumped from the Grøtsundet directly into the Centre for Marine Aquaculture. The water is aerated with oxygen before it enters the parental tanks in the brood fish hall. The seawater used for the egg incubators and larval tanks is furthermore filtered by a 90 µm drum filter, passes through a protein skimmer and a sand filter and is then UV treated before being used. Carbon dioxide concentrations in the acidified treatment were controlled by the semi-automated pH-Stat *IKS Aquastar* Systems, which activates magnetic valves to allow CO_2_ influx from a CO_2_ bottle. A pH sensor is attached to the outflow of the header tanks and if the pH is above a threshold, the system opens the valves in order to allow an inflow of CO_2_ in short pulses in order to maintain the seawater at the target value of 7.75. The pH was additionally checked daily with a WTW pH 3310 hand probe with a *SenTix*^®^ *H* pH-electrode. Water samples for carbonate chemistry were taken and analyzed at the University of Tromsø based on the Best Practices Guide (See Stiasny *et al*.^8^, for more details on the carbonate chemistry in the experiment).

### Parental treatment

Adult cod from the aquaculture stock of the company Nofima AS at the Centre for Marine Aquaculture in Tromsø, Norway were transferred from the sea cages to the centre on 16^th^ January 2014 to start the incubation. These aquaculture stocks were a F3 generation mixture of two wild stocks, the Norwegian coastal cod and the North-East-Arctic cod from the Barents Sea. They were kept in net cages in the fjord and were transferred using a well boat and transfer tanks. They were split into the two separate treatment tanks each of 4 m diameter filled with 18 m^3^ seawater containing about 80 fish each. The tanks had a constant seawater flow through of 225 l/minute. The light regime was matched weekly to outside conditions.

The adult cod were regularly checked for running eggs and sperm. When mature and running, they were strip-spawned and eggs were fertilized *in vitro*. Five non-siblings’ families from the ambient CO_2_ parental treatment and seven non-siblings’ families from the high CO_2_ parental treatment were used. Additionally, eggs from a natural spawning event were added to the ambient parental treatment, contributing 57% of the larvae in the ambient parent/ambient larvae treatment and 70% in the ambient parental/high CO_2_ larvae treatment. All eggs that were used were spawned on the same day.

### Egg and larval treatment

Fertilized eggs were transferred to incubators, which were kept at 6 °C and were constantly aerated. Families were kept in separate incubators, so that a balanced number of hatched larvae could be transferred at the start of the larval experiment. After hatching the larval density was assessed in the incubators by counting the number of larvae in five aliquots each of 100 ml and extrapolating the average number per ml to the volume of the incubator. 11 000 larvae were transferred into each rearing tank and the larval experiment was commenced. This day was set as 0 days post-hatching (dph), even though larvae had hatched over several days before. Each treatment combination was replicated in three separate larval tanks, which were randomly distributed within the larval rearing setup.

The larval tanks were initially maintained at 6 °C, but the temperature was later raised to 10 °C in all tanks to assist growth rates and survival^33^. The larvae were kept in light 24 hours a day. Larvae were fed with *Nannochloropsis* and *Brachionus* at different time intervals for the different food treatments (seven in the high compared to three times daily in the low food treatment). The prey concentrations given per feeding remained constant and the same for both treatments.

For more information on feeding concentrations, please consult the article and the SI of Stiasny *et al*.^8^.

### Survival measurements

Survival was measured three times in the larval tanks, on days 8, 12 and 16 post-hatching, by measuring the numbers of remaining larvae. Five subsamples of 0.8 l were taken across the whole water column using a pipe, which could be closed at the bottom, and the number of living larvae in the subsamples was counted. An increased aeration during the sampling process ensured an even distribution of larvae in the rearing tanks. The accuracy and precision of the method was repeatedly checked in separate tanks with a known number of larvae. After day 16 post-hatching of the experiment, the method became inaccurate and imprecise. This is likely due to the increased swimming ability of the larvae, combined with improving sensory abilities, which probably resulted in an uneven distribution of larvae in the tanks due to avoidance behaviour towards the pipe and the increased aeration. Survival data was therefore disregarded after 16 dph, but larvae were sampled for growth and histology measurements until 36 and 35 dph respectively.

### Growth

Ten larvae per tank were sampled at day 36 post-hatching, euthanized using Tricaine methanesulfonate (MS222), then frozen at −20 °C and later measured for Standard Length (mm) using calibrated digital images. In order to measure dry weight, larvae were freeze dried before being weighed.

### Statistical analysis

All statistical analyses were run in the program R (Version 3.3.2) and RStudio (Version 1.0.136). For growth measurements ten larvae per tank were sampled in order to get an accurate assessment of the variance in the tanks. To include the possibility of tank effects a linear mixed effects model (lme) was run to test for differences and interactions between the treatments, but also including the tank as a random factor. The dry weight was log transformed in order to achieve normality of residuals. Survival in percent was logit transformed before being assessed using a repeated-measures multi-factorial ANOVA including interactions between all treatments and across the three sampling days.

### Histology

Euthanized larvae were fixed in 4% buffered formaldehyde at 35 dph, embedded in *Technovit*^®^ or paraffin, sectioned transversely or longitudinally respectively at 3 µm, followed by staining with methylene blue (*Technovit* sections) or haematoxylin and eosin (paraffin sections)^34^. *Technovit*-sections from head region (with eyes, gills and heart), front part of gut (with liver, pancreatic tissue, kidney tissue) as well as paraffin sections were studied and photographed in the microscope (Leitz Aristoplan with a Leica DFC295 camera). Moderate or numerous amounts of vacuoles in the pigment layer of the retina were noted and given a subjective score from 0 to +++ (some-several-many) (see also Frommel *et al*.^6^). A similar score was used for registrations of lipid vacuoles in the cod larvae livers. The scoring was done by a single person (Inger-Britt Falk-Petersen) using repeated assessments.

The experiments were conducted at the Centre for Marine Aquaculture (formerly the National Cod Breeding Centre), NOFIMA, Tromsø, Norway in accordance to the national rules and regulations and all efforts where undertaken to minimize stress and suffering of the fish. The Norwegian Animal Research Authority (Forsøksdyrutvalget) approved the experiments (ethics permit number is FOTS ID 6382).

### Data availability

The datasets generated during and/or analysed during the current study will be available in the PANGEA repository.
